# The accuracy of echocardiography versus surgical and pathological classification of patients with ruptured mitral chordae tendineae: a large study in a Chinese cardiovascular center

**DOI:** 10.1186/1749-8090-6-94

**Published:** 2011-07-29

**Authors:** Weichun Wu, Xiaoliang Luo, Linlin Wang, Xin Sun, Yong Jiang, Shunwei Huo, Dalou Tu, Zhigang Bai, Hao Wang

**Affiliations:** 1 Department of Echocardiography, Cardiovascular Institute and Fuwai Hospital, Chinese Academy of Medical Sciences & Peking Union Medical College, Beijing 100037, China; 2 Department of Cardiology, Cardiovascular Institute and Fuwai Hospital, Chinese Academy of Medical Sciences & Peking Union Medical College, Beijing 100037, China; 3 Department of Pathology, Cardiovascular Institute and Fuwai Hospital, Chinese Academy of Medical Sciences & Peking Union Medical College, Beijing 100037, China; 4 Department of General Surgery, Beijing Friendship Hospital, Capital Medical University, Beijing 100050, China

**Keywords:** ruptured mitral chordae tendineae, echocardiography, surgery, pathology

## Abstract

**Background:**

The accuracy of echocardiography versus surgical and pathological classification of patients with ruptured mitral chordae tendineae (RMCT) has not yet been investigated with a large study.

**Methods:**

Clinical, hemodynamic, surgical, and pathological findings were reviewed for 242 patients with a preoperative diagnosis of RMCT that required mitral valvular surgery. Subjects were consecutive in-patients at Fuwai Hospital in 2002-2008. Patients were evaluated by thoracic echocardiography (TTE) and transesophageal echocardiography (TEE). RMCT cases were classified by location as anterior or posterior, and classified by degree as partial or complete RMCT, according to surgical findings. RMCT cases were also classified by pathology into four groups: myxomatous degeneration, chronic rheumatic valvulitis (CRV), infective endocarditis and others.

**Results:**

Echocardiography showed that most patients had a flail mitral valve, moderate to severe mitral regurgitation, a dilated heart chamber, mild to moderate pulmonary artery hypertension and good heart function. The diagnostic accuracy for RMCT was 96.7% for TTE and 100% for TEE compared with surgical findings. Preliminary experiments demonstrated that the sensitivity and specificity of diagnosing anterior, posterior and partial RMCT were high, but the sensitivity of diagnosing complete RMCT was low. Surgical procedures for RMCT depended on the location of ruptured chordae tendineae, with no relationship between surgical procedure and complete or partial RMCT. The echocardiographic characteristics of RMCT included valvular thickening, extended subvalvular chordae, echo enhancement, abnormal echo or vegetation, combined with aortic valve damage in the four groups classified by pathology. The incidence of extended subvalvular chordae in the myxomatous group was higher than that in the other groups, and valve thickening in combination with AV damage in the CRV group was higher than that in the other groups. Infective endocarditis patients were younger than those in the other groups. Furthermore, compared other groups, the CRV group had a larger left atrium, higher aortic velocity, and a higher pulmonary arterial systolic pressure.

**Conclusions:**

Echocardiography is a reliable method for diagnosing RMCT and is useful for classification. Echocardiography can be used to guide surgical procedures and for preliminary determination of RMCT pathological types.

## Introduction

Ruptured mitral chordae tendineae (RMCT) are increasingly reported as an important cause of mitral regurgitation (MR) [1], which is a progressive disease with severe clinical symptoms that eventually requires mitral valve (MV) surgery [2]. Valve repair and replacement are the currently accepted surgical treatments for severe MR [3]. Previous surgery, pathological changes and echocardiographic characteristics of the MV are reported to be associated with ruptured chordae tendineae[4-6], but no large-scale studies on the relationship between echocardiography, surgery and pathology have been reported. Furthermore, although echocardiography is a common tool for diagnosing RMCT, it is unclear how its accuracy compares with surgical findings and pathological classification of RMCT.

Therefore, we evaluated echocardiographic, surgical and pathological examinations of consecutive patients who underwent surgery for RMCT at Fuwai Hospital, which has a large cardiovascular center. Our study aimed to compare the accuracy of the preoperative predictive tests of thoracic echocardiography (TTE) and transesophageal echocardiography (TEE) with the gold standards of surgical findings and pathological examination.

## Materials and methods

### Patients and clinical characteristics

Echocardiographic, pathological and surgical findings were performed in 242 consecutive subjects who were in-patients for RMCT at Fuwai Hospital from January 1, 2002, to July 30, 2008. Most of the patients had chronic MR. The inclusion criteria were: 1) patients who underwent an operation; 2) diagnosis of RMCT was supported by surgical and pathological outcomes; 3) preoperative TTE was performed.

RMCT cases were classified by location as anterior or posterior, and by degree as partial or complete RMCT, according to surgical findings. RMCT cases were also classified by pathology into four groups: myxomatous degeneration, chronic rheumatic valvulitis (CRV), endocarditis, and others.

### TTE and TEE

TTE was performed in the left-lateral position using a commercially available machine (GE vivid 7, Phillips IE33) with a 3.5-8 MHz phased-array transducer. All patients underwent standard two-dimensional and Doppler echocardiographic examinations with detailed evaluation of heart function. Imaging planes were standardized, and they included the parasternal left heart long-axis view, the aortic and MV short-axis view, and the apical four- and two-chamber views.

Left atrial (LA) diameter was measured from the parasternal left heart long-axis view. Pulmonary artery trunk and pulmonary flow were measured from the aortic short-axis view. We also measured mitral inflow, including the E velocities and aortic valve flow. Pulmonary systolic pressure was calculated according to velocity of tricuspid regurgitation by the Bernoulli equation[7]. The left ventricular end-diastolic diameter (LVEDd) and ejection fractions (EF) were calculated by the M-mode method.

Valvular regurgitation was graded as: mild (I), which was defined as MR jets with an area < 20% of the LA area; moderate (II) as 20-40% of the LA area; and severe (III) as > 40% of the LA area [8]. Mild pulmonary artery hypertension was defined as a pressure of 36 to 51 mmHg [9].

TEE exams were usually conducted intraoperatively, using a GE vividI with a 12 MHz multiplane transesophageal transducer. The MV and its chordae tendineae were observed in the left ventricular midesophageal and MV transgastric views, with rotation of the TEE probe to achieve the clearest view.

### Histology and pathology

Sections of surgically excised tissues were paraffin-embedded, stained with hematoxylin and eosin for light microscopy, and reviewed at a minimum of four section levels by a cardiac pathologist who was blinded to the experimental status of each patient. Particular attention was given to recording primary microscopic features of the mitral leaflets and chordae tendineae, including fibrosis, degeneration, thickening, inflammatory changes and vegetation.

### Statistical analysis

Statistical analysis was performed with the SPSS 13.0 statistical software package. Continuous variables are presented as the mean ± standard deviation, with accounts and percentages as categorical variables. Differences between groups were analyzed using the chi-square test. TTE and TEE accuracy, and sensitivity and specificity for the detection of ruptured chordae were calculated according to standard formulae. The characteristics measured for different pathologies were compared using one-way ANOVA and S-N-K analysis. A P value ≤ 0.05 was considered statistically significant.

## Results

This study included 242 RMCT patients, with 178 males and 64 females, who were admitted to our hospital for MV surgery. The mean age was 50.63 ± 14.12 years (range, 7-81 years). All patients underwent TTE, and TEE was performed intraoperatively in 201 patients. Pathological analysis was performed in 171 patients. Electrocardiographic abnormalities were present in 193 patients, with 90 demonstrating atrial fibrillation. Patients were classified as functional class I to III by the New York Heart Association.

### Diagnostic accuracy of TTE and TEE compared with classification during surgery

Surgery was successfully performed for all patients and surgical findings revealed posterior leaflets (n = 148), anterior leaflets (n = 81) and rupture of both chordae tendineae (n = 13). Partial RMCT (n = 217) was more frequent than complete RMCT (n = 25). Sensitivity, specificity, positive and negative predictive values, and positive and negative likelihood ratios for TTE by surgical classification of RMCT patients are shown in Table 1.

**Table 1:** Sensitivity, specificity, positive and, negative predictive values, and positive and negative likelihood ratios for TTE by surgical classification of RMCT patients.

surgical classification	Sensitivity (%)	Specificity (%)	Positive predictive (%)	Negative predictive (%)	Positive Likelihood ratio	Negative Likelihood ratio
Anterior	92.6	96.9	93.8	96.3	29.8	0.08
Posterior	88.5	93.6	95.6	83.8	13.9	0.12
Complete	52.2	94.9	50.0	94.5	9.5	0.50
Partial	90.8	91.7	94.3	52.4	10.9	0.10

The diagnostic accuracy for RMCT was 96.7% for TTE and 100% for TEE compared with surgical findings. TTE showed a high sensitivity for diagnosing RMCT, except for complete RMCT, and a high specificity for diagnosing all types of RMCT. It also showed a very high positive likelihood ratio and low negative likelihood ratio for diagnosing most types of RMCT.

The surgical types included MV repair and replacement, and the methods of MV repair included leaflet resection, chordal shortening, and chordal transfer. The methods of MV replacement included a mechanical prosthetic valve and bioprosthetic valve. The method of choosing the type of surgical method depended on the location of the ruptured chordae tendineae (P < 0.01), but no relationship was observed between surgical method and complete or partial degree of RMCT (P > 0.05). Anterior leaflet RMCT had a higher valve replacement rate (n = 52, 64%), and posterior leaflets had a higher valve repair rate (n = 98, 66.2%) (Table 2).

**Table 2:** Surgical types of RMCT and relationship between surgical methods and location and degree of RMCT.

operation methods	location of RMCT*			degree of RMCT^#^	
	**Anterior**	**Posterior**	**both**	**Complete**	**Partial**

Valve replacement	52	50	8	13	97
Valve repair	29	98	5	12	120
Total number	81	148	13	25	217

*Chi-square = 23.72,P < 0.01

# Chi-square = 0.89,P > 0.05

### Echocardiography characteristics and their role in classification of RMCT pathology

Echocardiography characteristics were varied, so we assigned the commonly observed echocardiographic abnormalities into the following categories: direct signs, flail or whiplash valve motion (n = 210, 86.7%); MV prolapse (n = 242, 100%); MR, moderate (n = 72) to severe (n = 172); pulmonary artery hypertension, mild (n = 25), intermediate (n = 13) or severe (n = 6); left heart enlargement (n = 180), left and right heart enlargement (n = 30), left atrial enlargement only (n = 21), and left ventricular enlargement only (n = 4); heart function, six cases had EF values lower than 60% and the rest were greater than 60%; pleural effusion, which was observed in a few patients (n = 7); and other signs, including abnormal valve echoes and aortic regurgitation.

With regard to TTE indicators, the most common characteristics in our study were left heart enlargement, increased MV inflow and tricuspid regurgitation, and normal heart function.

**Figure 1:**
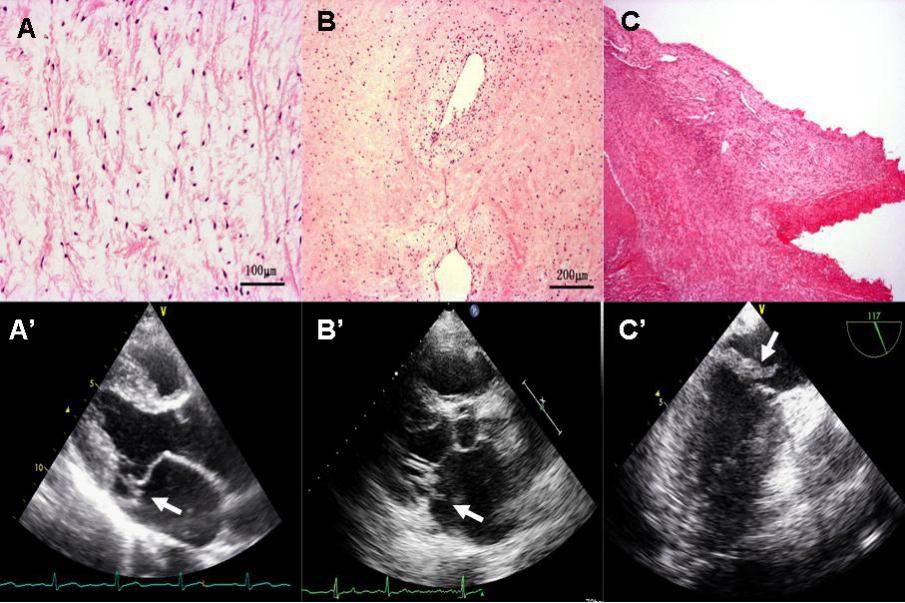
**Histological appearance and echocardiogram of the MV and chordae tendineae**. A: Myxomatous degeneration. The structure of the valve is crumbly and the main changes are myxoid degeneration and no inflammation. A': Myxomatous degeneration of idiopathic RMCT. The parasternal left ventricular long-axis view shows elongated subvalvular chordae, and a floppy and soft valve associated with posterior small tendon rupture. B: Chronic rheumatic valvulitis. Fibrous tissue hyperplasia of the valve, glass-like degeneration, and vascular proliferation, with a small amount of lymphocytic infiltration can be seen. B': Chronic rheumatic valvulitis. The parasternal left ventricular long axis view shows marked thickening of valve leaflets, and the arrow shows ruptured posterior tendons. C: Infective endocarditis. Valve tissue necrosis, thrombosis associated with a large amount of neutrophil infiltration, and neoplasms can be seen. C': Infective endocarditis (TEE): Intraoperative ultrasound shows marked thickening of mitral valve leaves and non-uniform, non-uniform echo dense and valve prolapse. The arrow indicates the site of chordae rupture and mitral valve prolapse.

The main pathological changes observed for RMCT were myxomatous degeneration (n = 96), chronic rheumatic valvulitis (n = 22), endocarditis (n = 10), and others (n = 43). Characteristics of TTE were different among the different pathological groups. The echocardiographic characteristics of RMCT included valvular thickening, extended subvalvular chordae, echo enhancement, and vegetation, as well as being combined with AV damage. The incidence of extended subvalvular chordae in the myxomatous group was higher than that in the other groups, and the incidence of valvular thickening combined with AV damage in the CRV group was higher than that in the other groups. Infective endocarditis patients were younger than those in the other groups, and there was a higher incidence of abnormal echo than in the other groups (Table 3 and Figure 1).

**Table 3:** Characteristics of TTE in the different pathological groups

Pathology	n	Age(Y)	valvular thickening	extended subvalvular chordae	Echo enhancement	Abonormal echo or Vegetation	combined with AV damage
myxomatous	96	54.24 ± 11.20	61(63.5)	74 (77.1) *	0	0	3(0.03)
CRV	22	51.93 ± 16.75	18(81.8) *	4(18.2)	6(27.3)	0	5(22.7) **
Endocarditis	10	38.20 ± 12.59**	6(60.0)	0	5 (50.0)	5 (50.0) *	0
others	43	52.33 ± 15.80	23(53.5)	10(23.3)	14(32.6)	7(16.3)	0

values are n (%). * P < 0.05, ** P < 0.01

With regard to structure and hemodynamic changes, compared other groups, the CRV group had a larger left atrium, higher aortic velocity, and higher pulmonary arterial systolic pressure (Table 4).

**Table 4:** Measurement and flow characteristics of TTE in the different pathological groups.

	Myxomatous	CRV	Endocarditis	others
LA	48.31 ± 9.37	52.00 ± 12.31*	43.50 ± 6.13	49.00 ± 8.81
LV	61.26 ± 6.79	61.09 ± 12.38	59.80 ± 8.65	61.83 ± 8.11
RV	20.57 ± 4.47	19.47 ± 5.91	19.60 ± 4.67	20.35 ± 6.22
AAO	30.63 ± 3.50	29.47 ± 6.56	26.75 ± 4.26	33.63 ± 3.38*
PA	25.00 ± 4.10	29.00 ± 4.21*	26.32 ± 2.78	26.67 ± 5.22
PV	0.85 ± 0.18	0.87 ± 0.21	0.84 ± 0.20	1.06 ± 0.67*
AV	1.19 ± 0.31	1.67 ± 1.12*	1.50 ± 0.12	1.26 ± 0.76
MV	1.61 ± 0.40	1.53 ± 0.48	1.71 ± 0.52	1.71 ± 0.30
TVR	3.47 ± 0.79	3.97 ± 0.59*	3.23 ± 1.05	3.38 ± 0.58
PASP	50.73 ± 22.11	64.32 ± 19.60*	43.56 ± 16.15	46.92 ± 14.96
EF%	66.03 ± 7.37	65.50 ± 7.93	67.30 ± 9.12	66.93 ± 7.02

Values are expressed as the mean ± S.D., Compared with other groups, * P ≤ 0.05

LA: left atrial; LV: left ventricle; RV: right ventricle; AAO ascending aorta; PA: Pulmonary artery trunk; PV: pulmonary flow velocity; AV: aorta flow velocity; MV: mitral flow velocity; TVR: tricuspid regurgitation velocity; PASP: pulmonary arterial systolic pressure MR: the degree of mitral regurgitation; EF: EF valve. CRV: chronic rheumatic valvulitis.

With regard to the relationship between pathology and location of RMCT, we found that posterior chordae tendineae rupture of the MV in myxomatous degeneration was greater than that in the other groups. Anterior chordae tendineae rupture of the MV was common in chronic valvulitis and infective endocarditis patients, while in the others groups had mainly posterior chordae tendineae rupture (Table 5).

**Table 5:** Relationship between pathology and location of RMCT.

	Myxomatous	CRV	Endocarditis	others	total
Anterior	22	14	7	11	54
Posterior	70	7	2	31	110
both	4	1	1	1	7
total	96	22	10	43	171

Chi-squareã23.97, P = 0.001

CRV: chronic rheumatic valvulitis

## Discussion

RMCT is a well known cause of serious MR [10] and usually requires surgery. We compared surgical findings and pathological examinations with echocardiographic examinations in a large series of RMCT patients, with the goal of determining general relationships between these factors.

Analysis of the clinical and echocardiographic characteristics of the patient cohort showed more male patients than females. All patients were surgical cases with MR, which was rated moderate to severe, and it resulted in an increase in the left ventricular volume that accelerated mitral flow velocity and enlarged the left heart chamber. As a result of long-term MR, some degree of high pulmonary arterial pressure was observed, and it was mainly mild to moderate. Cardiac function was normal for most patients, with lower cardiac function associated with a greater risk for valve replacement surgery. Direct and typical signs of RMCT were chain-flail or whiplash-like changes, which had an incidence of 86.7%, consistent with some reports that mitral chord rupture is the leading cause of mitral leaflet flail [11]. When these signs were not observed, most cases were second or third level tendon ruptures that were confirmed by surgery.

### Echocardiography versus surgical findings in the classification of RMCT

Using surgery as the gold standard, echocardiography was found to be an accurate method for diagnosing RMCT, and TEE showed a higher diagnostic accuracy than that for TTE, which is consistent with previous studies [12]. mainly because transesophageal echocardiography was not only closing to heart, high frequency but also performed on a sedated patient and examiner may be more experience. However, TTE still has a high diagnostic accuracy rate (96.7%) and has simple, convenient and noninvasive features. Furthermore, echocardiography accurately classified the site and degree of ruptured tendons. Preliminary experiments demonstrated that the sensitivity and specificity of diagnosing anterior, posterior and partial RMCT were very high, but the sensitivity of diagnosing complete RMCT was low. The reason for this finding may be because part of the small tendons under the flap could not be viewed or prudently diagnosed. Posterior chordae tendineae rupture of the MV was the most common finding, which might be because the posterior leaflet chordae were thinner, and they failed under less strain and load than those of anterior leaflet chordae; therefore, failure was most common for the posterior marginal chordae [1].

Our results indicate that echocardiography can be used to guide surgeons in choosing a method of operation. Some studies have shown that the surgical methods used depend on the location of the ruptured chordae tendineae[13]. The repair rate for the RMCT posterior leaflet was higher than that for the anterior leaflet, possibly because the MV posterior lobe ring circumference is approximately two-thirds longer, and therefore, the ring was often simply shortened for repair. Furthermore, posterior RMCT occurred more frequently with myxomatous degeneration and anterior RMCT was common in CRV and IE patients, which could be another possible reason for choosing difference operation methods. Additionally, the location of chordae tendineae rupture or prolapse may affect the survival of patients with MV repair, because Dania et al reported that reoperation was required after repair or replacement, but it was more frequent after repair of anterior MVP[14].

Our data showed that the surgical success rates for complete and partial RMCT were not significantly different. With improved surgical techniques, such as the implantation of artificial chordae tendineae, the rate of replacement in complete RMCT has been greatly reduced [15,16].

In the current study, on the basis of TTE and TEE evaluation, the majority of patients with RMCT had successful valve repair or replacement. Echocardiography is a powerful tool to define the mechanisms of RMCT and to identify the suitability of patients for a valve operation.

### Echocardiography versus pathology examination in the classification of RMCT

Our study found mild valve leaflet thickening and extensive subvalvular chordae in more than half of RMCT patients, representing almost all pathological types (Figure 1). Echocardiography identified that mild valve leaflet thickening accounted for 58.7%, and extensive subvalvular chordae for 53.8% of cases, which may explain why leaflet thickness and length were closely related to the occurrence of RMCT or severe MR [17]. Echo enhancement and abnormal echo were also very common in all pathological types.

We were able to make a preliminary classification of pathology type using echocardiography characteristics. Our results were consistent with our previous study that myxomatous degeneration was the most common reason that causes mitral regurgitation [15]. In the current study, myxomatous degeneration showed significantly extended and floppy characteristics in 77.1% of this kinds of patients. According to Gabbay et al, although subacute infective endocarditis (SBE) and CRV have sharply dropped to 37.4% and 24.8%, respectively, since 1985, they are still considerable causes of RMCT [18]. In the current study, CRV has the characteristics of valve thickening (81.8%) and being combined with AV damage (22.7%). Furthermore, we found that it had a larger left atrium, higher aortic velocity, and higher pulmonary arterial systolic pressure, probably because of mitral stenosis and AV damage by rheumatism[19]. We also found that endocarditis had more unique echo characteristics in all pathological groups. The defining feature of endocarditis was vegetation, most of which is polypoid[20].

Others pathology groups included mainly fibroid degeneration, myxomatous with fibroplasia degeneration or chronic valvulitis. The last two types are rare in RMCT pathology. They all lacked specific echo manifestation.

Furthermore, according to our study, different pathologies had a rupture in different parts of the chordae tendineae, and this was may be one of the reasons why MV repair was suitable for myxomatous degeneration[21] and MV was suitable for replacement for chronic rheumatic valvulitis and infective endocarditis.

## Conclusions

echocardiography was found to be a reliable method for diagnosing RMCT with a high accuracy, and it played an important role in classification. This large RMCT study on echocardiography, surgery and pathology provided characteristics and details of RMCT. We found that we could use pre-operative echocardiographic RMCT to guide surgical procedures and determine possible pathological types.

## Competing interests

The authors declare that they have no competing interests.

## Authors' contributions

WWC, LXL and WLL participated in the design and coordination of the study, HSW and TDL participated in the data collection, SX and JY revised the manuscript, WH and BZG performed the statistical analysis and helped to draft and revise the manuscript. All authors read and approved the final manuscript.
